# Development of a novel patient-oriented tool to assess achalasia symptoms and response to treatment (I-PASS, International Patient-oriented tool for Achalasia Symptom Score)

**DOI:** 10.1093/dote/doaf114

**Published:** 2025-12-11

**Authors:** Giovanni Zaninotto, Sheraz R Markar, Rami Sweiss, Renato Salvador, Lorena Torroni, Francesco Casella, Humayra Dervin, Andrea Costantini, Sara Jamel, Michele Sacco, the I-PASS Working Group

**Affiliations:** Department of Surgery and Cancer, Imperial College London, London, UK; Nuffield Department of Surgical Sciences, University of Oxford, UK; Gastroenterology, University College London Hospitals NHS Foundation Trust, London, UK; Department of Surgical, Oncological, and Gastroenterological Sciences, School of Medicine, University of Padua, Padova, Italy; Department of Medicine, Saint Camillus International University of Health and Medical Sciences, Rome, Italy; Unit of Epidemiology and Medical Statistics, Department of Diagnostics and Public Health, University of Verona, Verona, Italy; Upper GI Surgery Division, University of Verona, Verona, Italy; Gastroenterology, University College London Hospitals NHS Foundation Trust, London, UK; Department of Surgical, Oncological, and Gastroenterological Sciences, School of Medicine, University of Padua, Padova, Italy; Department of Surgery and Cancer, Imperial College London, London, UK; Upper GI Surgery Division, University of Verona, Verona, Italy

**Keywords:** achalasia, benign esophageal disorder, patient-reported outcome

## Abstract

Achalasia treatment outcomes are often assessed using the Eckardt score, which has not been formally validated and is not designed as a patient-reported outcome (PRO) tool. To address this unmet clinical need, a group of healthcare providers developed the International Patient-oriented tool for Achalasia Symptom Score (I-PASS), which aims to record both symptom severity and frequency in a patient-oriented manner. Using the RAND/ University of California, Los Angeles, Appropriateness Methodology, a 16-member international, multidisciplinary panel identified key symptomatic domains through three Delphi rounds. Dysphagia, regurgitation (daytime and nocturnal), and chest pain were selected as primary domains, with weight loss included as an additional factor. Severity and frequency scoring were agreed upon, resulting in a maximum composite score of 53. The pretreatment I-PASS was pilot-tested in 118 treatment-naïve achalasia patients in the UK and Italy. Comprehension, completion time, acceptability, and correlation with the standard Eckardt score were assessed. All the patients completed the I-PASS. Most (96.5%) reported full comprehension, the median completion time was 10 minutes, and 98% expressed willingness to complete it again. The median I-PASS score was 29 (IQR 20–36), compared with a median Eckardt score of 7 (IQR 5–9). A strong correlation was observed between I-PASS and Eckardt (*ρ* = 0.68, *P* < 0.0001). Regression analysis confirmed that each one-point increase in Eckardt corresponded to a 3.1-point increase in I-PASS. The I-PASS questionnaire is feasible, well accepted, and provides a more comprehensive assessment of achalasia symptoms than the Eckardt score. Future studies will evaluate its reliability, responsiveness, and validity as a standardized PRO instrument.

## INTRODUCTION

Achalasia is a rare disorder characterized by a poorly relaxing lower esophageal sphincter and a loss of peristalsis (types I and II) or spastic contraction (type III). This results in impaired passage of food and liquids through the cardia and causes symptoms such as dysphagia, regurgitation, or chest pain. Treatments for achalasia aim to disrupt the nonrelaxing lower esophageal sphincter, through either forceful pneumatic dilation, myotomy at the cardia (performed endoscopically or via minimally invasive surgery), or temporarily paralyzing the muscle with botulinum toxin injections.^1^^,^^2^ The clinical success of these treatments is usually assessed by measuring symptom improvement, most often using the Eckardt score, which evaluates dysphagia, regurgitation, chest pain, and weight loss. The first three components are scored from 0 to 3 based on frequency, while weight loss is scored from 0 to 3 depending on the amount lost (no loss, <5 kg, 5–10 kg, >10 kg). Patients are classified as having a good outcome if their Eckardt score is less than 3, or a poor outcome if it is greater than 3.^3^ The International Society for Diseases of the Esophagus (ISDE) guidelines for esophageal achalasia note that, although the Eckardt score is widely utilized, it has never been validated beyond comparison with physiological measures, it may be influenced by healthcare professionals’ biases during patient interviews, and the chest pain and weight loss components could reduce the score’s reliability and validity.^4^^,^^5^ These observations highlight a crucial gap in accurately measuring symptoms before and after treatment, underscoring the need for a well-designed, patient-oriented instrument suitable for application in both real-world clinical practice and randomized controlled trials.

This study aimed to develop and conduct a preliminary test of a pretreatment achalasia symptom questionnaire, evaluating patients’ acceptance of the questionnaire and their willingness to complete it independently. The secondary aims included assessing the time taken by patients to complete the questionnaire, measuring patient satisfaction, and examining the correlations between the questionnaire responses and those from the traditional Eckardt score.

## METHODS

We designed a two-step study: in the first step, we developed the symptom questionnaire using the Delphi methodology, and in the second step, we piloted the questionnaire with a group of naïve achalasia patients.

### Development of the pretreatment questionnaire

An international, multidisciplinary team of 8 surgeons, 7 gastroenterologists, and 1 clinical psychologist (5 from the USA/Canada, 10 from Europe, and 1 from Australia) collaborated to reach a consensus on the symptoms to include in the questionnaire and how to grade their severity and frequency.

The selection criteria for the working group included expertise in esophageal diseases, peer-reviewed publications in the field, and representation from various practice and geographical settings.

The employed method was the RAND/University of California, Los Angeles Appropriateness Methodology (RAM) to assess agreement with each statement. The initial list of domains was created by the Convenors (G.Z. and S.R.M.) through a comprehensive review of the literature and interviews with patients, and it included nine disease-specific impact domains: Dysphagia, Regurgitation, Chest Pain, Weight Loss, Reflux, Heartburn, Nocturnal Cough, Hypersalivation, and Social Embarrassment.

We asked the panelists to choose the primary symptoms to include in the questionnaire and to rate them from 1 (most relevant) to 9 (least relevant). We inquired about the ideal time frame for assessing frequency (the day before, the past week, the previous two weeks, the last month) and how to evaluate the severity of each symptom. We specifically asked which methodology was best for assessing chest pain (whether on a discrete or continuous scale), measuring weight loss, and whether it was necessary to consider recording additional factors such as chewing speed and the time taken to finish a meal. The Delphi process was conducted via mail in three separate stages. We retained statements with an approval rate exceeding 80% and a final ranking with a median score of 4, and after each stage, the panelists re-ranked the questions that did not reach a consensus.

Two English achalasia patients helped us translate the final version of the I-PASS questionnaire into plain language, rephrasing medical terms such as ‘dysphagia’ as ‘trouble swallowing’, ‘regurgitation’ as ‘undigested food or liquid coming back into your throat after eating’, and ‘chest pain’ as ‘sensation of sharp stabbing or burning pain, a dull ache, pressure, or tightness in the chest, behind the breastbone, or in the back’.

The draft questionnaire was presented and informally discussed with members of Achalasia Action during a meeting held in September 2019 in London (Achalasia Action is a UK patient support charity, formed almost exclusively by achalasia patients or patient advocates).

The English version of the I-PASS questionnaire was translated into Italian and then back-translated by a native professional translator. Two Italian achalasia patients also reviewed the questionnaire and adapted it into lay terms. The I-PASS questionnaire is available in the supplementary section.

For each symptom, specific questions were devised and assigned scores for both severity and frequency. For instance, in the case of dysphagia, the question ‘Do you have any trouble swallowing?’ allows for graduated responses, with increasing scores depending on severity (from 0 for ‘No swallowing trouble’ to 4 for ‘I have trouble swallowing both liquid and solid food’). Similarly, the symptom frequency was scored from 0 (never) to 5 (at every meal or daily episode). As with other clinical questionnaires, we decided to calculate each partial score by multiplying severity and frequency, since this method better captures the overall symptom burden, preventing the underestimation of infrequent but severe manifestations or the overestimation of frequent but mild ones. The total score is obtained by summing the maximum scores for each symptom (20 for dysphagia, 10 for daytime regurgitation, 8 for nighttime regurgitation, 12 for chest pain, and 3 for weight loss), resulting in a total of 53. Weight loss is measured directly and scored as a single value (based on the amount of kilograms lost: <5 kg earns 1 point, 5–10 kg earns 2 points, >10 kg earns 3 points), with no weight loss earning a score of 0 (Table 1).

**Table 1:** Structure and scoring of the I-PASS questionnaire: assessment of symptom severity in patients with achalasia^†^

	**Severity**		**Pts**	**Frequency**		**Pts**
**Dysphagia**	*Do you have any trouble swallowing?*	No	0	I never experience any trouble swallowing		0
		I have regular trouble swallowing only certain food	1	I have trouble swallowing only once/twice a month		1
		I have regular trouble swallowing solid food	2	I have more frequent trouble swallowing	I have trouble swallowing once a week	2
		I have regular trouble swallowing liquid	3		Every 2 to 3 days, or more	3
		I have regular trouble swallowing both liquid and solid food	4		At least once, every day	4
					At every meal	5
**Subtotal**						**20**
**Daily Regurgitation**	*Do you regularly have undigested food or liquid coming back into your throat after eating?*	No	0	I never experience the food/liquid coming back into my throat or mouth		0
		Yes	2	I experience the food/liquid coming back into my throat or mouth occasionally (once or twice every month)		1
				I experience the food/liquid coming back into my throat or mouth more frequently	Once a week	2
					Every 2 to 3 days or more	3
					Every day (at least one episode)	4
					Every meal	5
**Subtotal**						**10**
**Regurgitation at night**	*Do you regularly have food or liquid coming back into your mouth at night or when you lie down?*	No	0	I never experienced undigested food or liquid coming back during the night		0
		Yes	2	It happens only occasionally (once/twice per month)		1
				It happens more frequently	Once a week	2
					Every 2 to 3 nights or more	3
					Every night (at least one episode)	4
**Subtotal**						**8**
**Chest Pain**	*Could you please define the severity of your chest pain?*	I never experienced chest pain	0	I never experience chest pain		0
		My chest pain is mild, I just need to take a deep breath or swallow some liquid	1	I experience chest pain only occasionally (once or twice per month)		1
		I need to take a pain killer or other medication	2	I experience chest pain more frequently	Once a week	2
		I have needed to rest in bed or visit the emergency room/hospital/clinic	3		Every two to three days	3
					Every day (at least one episode)	4
**Subtotal**						**12**
**Weight Loss**	*Did you lose weight in the past 6 months?*	No		If you lost weight, how many kilos did you lose? (kg)‡	No	0
		Yes			<5	1
					5–10	2
					>10	3
**Sub-total**						**3**
**TOTAL SCORE** ^*^						**53**

^†^The table shows the domains, associated questions, and response modes of the I-PASS questionnaire, used to quantify the severity of achalasia symptoms in patients. Each domain provides an assessment of symptom severity and frequency, with scores assigned to each response mode. ^*^The total I-PASS score (range 0–53) is obtained by summing the products between the severity and frequency scores for each symptom within each domain. A higher score indicates greater overall disease severity.

### Pilot testing of the pretreatment I-PASS questionnaire

For this pilot study, we administered the pretreatment questionnaire to naive patients with achalasia confirmed through high-resolution manometry and barium swallow, while an endoscopy ruled out malignancies in the esophagus and stomach. The exclusion criteria included being under 18 years of age, prior treatment, lack of fluency in English or Italian, neurological impairments or significant psychiatric disorders, and incomplete high-resolution manometry studies. Consent to participate was obtained from all the patients. Patients completed the I-PASS questionnaire and the SF-36 quality-of-life questionnaire independently, although research team members were available to provide further explanations if needed. The time taken to complete the questionnaires was recorded. Separately, a healthcare professional interviewed each patient and completed the Eckardt score. Finally, patients were asked to indicate on an analog scale their level of comfort completing the I-PASS questionnaire, whether they understood all the questions, whether any were unclear, and whether they would be willing to complete the questionnaire again after treatment.

## STATISTICS

All the anonymized patient data and questionnaires were recorded in a case report form and transferred to an electronic database (REDCap).

The data were presented as frequencies and percentages for categorical variables and as medians with interquartile ranges (p25–p75) for non-normally distributed continuous variables. The distribution of variables was assessed using the Shapiro–Wilk normality test.

We evaluated the I-PASS score using Spearman’s correlation coefficient to compare it with the Eckardt scores. Simple linear regression was used with I-PASS as the dependent variable and Eckardt’s score as the independent variable to assess the linear relationship and predictive effect of Eckardt’s score on the I-PASS score (regression coefficient, 95% confidence interval, and significance).

A box plot and the nonparametric Kruskal–Wallis test assessed whether I-PASS increased consistently with the rise of the Eckardt score. The relationship between I-PASS scores and the Eckardt score was analysed using the Bland–Altman method.

We used version 18 of STATA software (StataCorp, College Station, TX, USA) for statistical analysis; statistical significance was set at *P* < 0.05.

## ETHICS

The part of the study involving patients was approved by the Health Research Authority and Health and Care Research Wales (protocol number 19SM5619, October 2020), the Imperial College NHS Trust Ethical Committee (n.20/EM/0167), the University College London Hospitals NHS Foundation Trust (Ref. 135152—IRAS 267141, 07/04/2021), and the Ethical Committee of the Verona University Hospital (Prot n. 77798, 27/12/2022).

The study was funded by the Fondazione Morgagni and endorsed by the Achalasia Patient Association in the UK and ALMA (Associazione Libera Malati di Acalasia) in Italy.

## RESULTS

### Expert consensus

An almost unanimous consensus was reached on four symptom domains: dysphagia, regurgitation, chest pain, and weight loss. After each step, the panel members re-evaluated the possible answer choices regarding severity, frequency, and the relevant time window. Concerning dysphagia, we agreed to document its occurrence with various foods, including rice, bread, apples, and meat, and to score its severity if it occurred with liquids, solid foods, or both. We also agreed to record instances of both postprandial and nocturnal regurgitation, and to classify the severity of chest pain based on the need for simple maneuvers (such as taking a deep breath or sipping water), or for painkillers, and whether bed rest or an emergency visit was necessary. The panel members agree on a time window of a week before answering the questionnaire, and 6 months for weight loss. The final questionnaire comprised seven items with 42 possible answer choices. (The I-PASS questionnaire is available in the Supplementary material).

### Pilot testing of the pretreatment I-PASS questionnaire

As of July 2024, we had tested the I-PASS pretreatment questionnaire on 118 naïve achalasia patients, of whom 62 were women (52%), with a median age of 52 years (range 41–60). Eighteen patients were recruited in the UK (UCLH), while 100 were enrolled in Italy at the University of Padova and the University of Verona Hospitals. All UK patients were on a waiting list for endoscopic myotomy (POEM), whereas all patients in Italy were awaiting laparoscopic Heller-Dor surgery.

All the patients completed the questionnaire. Ninety-six per cent understood all the questions, with only four patients (three in Italy and one in the UK) failing to comprehend one or more questions. In comparison, 10 patients had difficulty understanding some questions within the validated SF-36 questionnaire, while one struggled with a few questions in the Eckardt score. (see in Supplementary Tables 2 and 3). The median time to complete the I-PASS questionnaire was 10 minutes (IQR 5–15). One hundred and eleven patients (98%) confirmed their willingness to complete the I-PASS questionnaire again after treatment.

The median I-PASS score was 29 (range 1–50; IQR 20–36), with no one reaching the maximum score of 53. The median Eckardt score was 7 (range 1–12; IQR 5–9) (Supplementary Fig. 1).

There was a strong positive correlation between the I-PASS and the Eckardt score, with a Spearman’s rho of 0.681 (*P* < 0.0001). This result suggests that patients with higher Eckardt scores also tended to report higher I-PASS scores, confirming the consistency between the two measures.

We calculated the regression coefficient between the two scores, with the I-PASS as the dependent variable and the Eckardt score as the independent variable. On average, every 1-point increase in the Eckardt score corresponded to an increase of 3.11 points in the I-PASS. The narrow confidence interval and significance reinforce the robustness of this linear relationship (95% CI 2.445–3.786, *P* < 0.001), although this analysis was not adjusted for potential confounders.

The agreement between the two methods was further assessed using the Bland–Altman approach, which confirmed that, beyond their correlation, the two scores also demonstrated good agreement. In other words, the I-PASS and Eckardt Score yielded largely overlapping results for most patients.

## DISCUSSION

This study outlines the development and initial evaluation of the International Patient-oriented Achalasia Symptom Score (I-PASS), the first tool specifically designed to measure both the frequency and severity of achalasia symptoms from the patient’s perspective. Created through an international Delphi consensus and refined with patient lay-term adaptation, I-PASS provides a more detailed and patient-driven alternative to the Eckardt score.

The questionnaire demonstrated high feasibility: nearly all the patients completed it independently, understanding was excellent, and the median completion time was short (10 minutes). The willingness of almost all the participants to repeat the questionnaire indicates that I-PASS is suitable for both clinical practice and research. Importantly, I-PASS showed a strong correlation with the Eckardt score, supporting its preliminary construct validity and providing more detailed symptom assessment. Unlike the Eckardt score, which depends solely on frequency, I-PASS also measures severity and distinguishes between daytime and nocturnal regurgitation, a clinically significant difference.

These findings indicate that I-PASS could be a useful tool to standardize outcome reporting in achalasia. By allowing patients to complete the questionnaire themselves prior to consultation, I-PASS reduces investigator bias and provides clinicians with a structured and comprehensive symptom profile.

### Limitations

This study has several notable limitations. Although the Delphi process identified key domains and structured the scoring system, we did not conduct qualitative interviews or focus groups with patients to generate items based on their personal experiences. Similarly, no formal cognitive interviewing or pretesting was undertaken with draft items to confirm understanding and relevance, aside from the small group of patients involved in lay-term adaptation. We did, however, have a thorough but informal discussion with the Achalasia Action members during the meeting when the questionnaire was presented. The delays in these steps were caused by the COVID-19 pandemic and will be addressed in future research.^6^ Second, this pilot phase did not include formal psychometric testing; internal consistency (e.g. Cronbach’s *α*), test–retest reliability, and construct validity remain unassessed. Third, we did not examine responsiveness or the ability to detect change over time, such as the capacity of the I-PASS to identify meaningful clinical improvements following therapy. Lastly, the current study focuses on pretreatment assessment, so the utility of posttreatment evaluation will need to be verified in future prospective cohorts.

Future directions will therefore include qualitative research, cognitive interviewing, and comprehensive psychometric validation across multiple centres and achalasia subtypes. These steps will determine whether I-PASS can reliably measure treatment response and act as a standardized PRO in both clinical practice and trials.

## CONCLUSION

The I-PASS questionnaire, developed by healthcare providers, is a new, patient-focused tool for assessing and measuring symptoms of achalasia. Pilot testing shows that it is practical and well accepted across various cultural contexts, providing a foundation for further validation with a larger patient group and in different settings.

## Supplementary Material

Fig_1_Supplementary_material_doaf114

Table_2_and_3_Supplementary_material_copy_doaf114

## Notes

Shanda H Blackmon, Guy Boeckxstaens, Ines Gockel, Donald Low, Eivind Ness Jensen, Joel E Richter, Renato Salvador, Giovanni Sarnelli, Edoardo Savarino, Daniel Sifrim, Rami Sweiss, Tiffany H Taft, Anders Thorell, David R Urbach, David Watson, Giovanni Zaninotto. DeBakey Department of Surgery, Baylor College of Medicine, Houston, TX, USA; Department of Chronic Diseases and Metabolism-Translational Research in Gastrointestinal Disorders, University of Leuven, Leuven, Belgium; Upper GI Surgery, Clarunis, University Digestive Health Care Center, Basel, Switzerland; Digestive Disease Institute Esophageal Center of Excellence, Virginia Mason Medical Center, Seattle, WA, USA; NTNU, Norwegian University of Science and Technology and Nord-Trøndelag Hospital Trust, Levanger, Norway; Department of Internal Medicine – Morsani College of Medicine, University of South Florida, Tampa, FL, USA; Dept of Surgery, Oncology and Gastroenterology, University of Padua, Padua, Italy; Department of Clinical Medicine and Surgery, University of Naples ‘Federico II’ Naples, Italy; Wingate Institute of Neurogastroenterology, Queen Mary University of London, London, UK; Department of Gastroenterology, University College London Hospitals NHS Foundation Trust, London, UK; Nortwestern University, Chicago IL, USA and The Rome Foundation Research Institute, Chapel Hill, NC, USA; Karolinska Institutet, Stockholm, Sweden; Temerty Faculty of Medicine, University of Toronto, Toronto, ON, Canada; Flinders Health & Medical Research Institute, Flinders University, Adelaide, SA, Australia; Department of Surgery and Cancer, Imperial College London, London, UK.
