# Supplementary material for: Development of a novel patient-oriented tool to assess achalasia symptoms and response to treatment (I-PASS, International Patient-oriented tool for Achalasia Symptom Score)
Source: Dis Esophagus. 2025 Dec 11;38(6):doaf114. doi: 10.1093/dote/doaf114 (PMC12696712; doi:10.1093/dote/doaf114)
Supplement: Fig_1_Supplementary_material_doaf114 [file fig_1_supplementary_material_doaf114.docx]

**Fig 1.**

| Score | Median | IQR | Min-max |
| --- | --- | --- | --- |
| Eckardt | 7 | 5-9 | 1-12 |
| I-PASS | 29 | 20-36 | 1-50 |

**Legend to Figure 1**

Distribution of I-PASS and Eckardt scores
