# Supplementary material for: Development of a novel patient-oriented tool to assess achalasia symptoms and response to treatment (I-PASS, International Patient-oriented tool for Achalasia Symptom Score)
Source: Dis Esophagus. 2025 Dec 11;38(6):doaf114. doi: 10.1093/dote/doaf114 (PMC12696712; doi:10.1093/dote/doaf114)
Supplement: Table_2_and_3_Supplementary_material_copy_doaf114 [file table_2_and_3_supplementary_material_copy_doaf114.docx]

T**able 2: I-PASS pre-treatment questionnaire**

|  | Summary (n=118) | |
| --- | --- | --- |
| Sex | |  |
| Woman | | 61 (51.7%) |
| Man | | 57 (48.3%) |
| Age* | | 52 (41-60) |
| Do you have any trouble swallowing? | |  |
| No | | 3 (2.5%) |
| I have regular trouble swallowing only certain food | | 6 (5.1%) |
| I have regular trouble swallowing solid food | | 32 (27.1%) |
| I have regular trouble swallowing liquid | | 4 (3.4%) |
| I have regular trouble swallowing both liquid and solid food | | 73 (61.9%) |
| The frequency of your trouble swallowing? | |  |
| I never experience any trouble swallowing | | 3 (2.5%) |
| I have trouble swallowing only once / twice a month | | 1 (0.8%) |
| I have more frequently trouble swallowing | | 114 (96.6%) |
| If your swallowing problem (of any severity) is more frequent, please consider the last week: | |  |
| I have trouble swallowing once a week | | 4 (3.5%) |
| Every two to three days, or more | | 10 (8.8%) |
| At least once, every day | | 25 (22.1%) |
| At every meal | | 74 (65.5%) |
| Do you regularly have undigested food or liquid coming back into your throat after eating? | |  |
| Yes | | 108 (91.5%) |
| No | | 10 (8.5%) |
| Do you regularly have food or liquid coming back into your mouth at night or when you lay down? | |  |
| Yes | | 109 (92.4%) |
| No | | 9 (7.6%) |
| When the food or liquid comes back into your throat or mouth, what do you do? | |  |
| I typically swallow back the food/liquid | | 30 (28.3%) |
| I typically spit it out | | 76 (71.7%) |
| Frequency of regurgitation? | |  |
| I never experience the food/liquid coming back into my throat or mouth | | 16 (13.6%) |
| I experience the food/liquid coming back into my throat or mouth occasionally (once or twice every month) | | 20 (16.9%) |
| I experience the food/liquid coming back into my throat or mouth more frequently | | 82 (69.5%) |
| If the food/liquid comes back up more frequently, please consider the last week: | |  |
| Once a week | | 6 (8.3%) |
| Every two to three days or more | | 14 (19.4%) |
| Every day (at least one episode) | | 29 (40.3%) |
| Every meal | | 23 (31.9%) |
| The frequency of undigested food/liquid coming back into your mouth at night or when you lay down: | |  |
| I never experience undigested food or liquid coming back during the night | | 26 (22.2%) |
| It happens only occasionally (once/twice per month) | | 12 (10.3%) |
| It happens more frequently | | 79 (67.5%) |
| If the return of undigested food or liquid into the throat or mouth occurs more often, please consider the last week: | |  |
| Once a week | | 15 (19.2%) |
| Every two to three nights or more | | 37 (47.4%) |
| Every night (at least one episode) | | 26 (33.3%) |
| Could you please define the severity of your chest pain? | |  |
| I never experienced chest pain | | 19 (16.4%) |
| My chest pain is mild, I just need to take a deep breath or swallow some liquid | | 60 (51.7%) |
| I need to take a pain killer or other medication | | 26 (22.4%) |
| I have needed to rest in bed or visit the Emergency Room/ Hospital /Clinic | | 11 (9.5%) |
| Frequency of the chest pain ? | |  |
| I never experience chest pain | | 20 (17.1%) |
| I experience chest pain only occasionally (Once-twice per month) | | 36 (30.8%) |
| I experience chest pain more frequently | | 61 (52.1%) |
| If your chest pain occurs more frequently, please consider the last week it has occurred: | |  |
| Once a week | | 16 (27.1%) |
| Every two to three days | | 24 (40.7%) |
| Every day (at least one episode) | | 19 (32.2%) |
| Current weight (kg)* | | 67 (57-78) |
| Current height (cm)* | | 170 (165-178) |
| Did you lose weight in the past 6 months? | |  |
| Yes | | 89 (75.4%) |
| No | | 29 (24.6%) |
| If you lost weight, how many Kg did you loose? (kg)* | | 5 (1-10) |

*Median, p25-p75

**Table 3 : Summary of satisfaction questionnaire.**

|  | **Summary (n=118**) |
| --- | --- |
| **Did you understand all the questions in the questionnaires?** |  |
| Yes | 110 (96.5%) |
| No | 4 (3.5%) |
| **If some questions were unclear, which questionnaires were they in?** |  |
| I-pass | 13 (54.2%) |
| SF 36 | 10 (41.7%) |
| Eckardt Score | 1 (4.2%) |
| **Could you please tick on this analogic scale how comfortable you felt in filling** |  |
| 2 | 3 (2.7%) |
| 3 | 1 (0.9%) |
| 5 | 3 (2.7%) |
| 6 | 4 (3.5%) |
| 7 | 11 (9.7%) |
| 8 | 26 (23.0%) |
| 9 | 22 (19.5%) |
| 10 | 43 (38.1%) |
| **Will you be available to answer these questionnaires again after your treatment?** |  |
| Yes | 111 (98.2%) |
| No | 2 (1.8%) |
| **Time needed to complete the I-PASS questionnaire: (mins)** | 10 (5-15) |
